# GLASS Clinical Decision Rule Applied to Thoracolumbar Spinal Fractures in Patients Involved in Motor Vehicle Crashes

**DOI:** 10.5811/westjem.2017.7.34157

**Published:** 2017-09-21

**Authors:** Seth Althoff, Ryan Overberger, Mark Sochor, Dipan Bose, Joshua Werner

**Affiliations:** * Einstein Medical Center, Department of Emergency Medicine, Philadelphia, Pennsylvania; † University of Virginia, Department of Emergency Medicine, Charlottesville, Virginia; ‡ World Bank, Transport Specialist, Washington, DC

## Abstract

**Introduction:**

There are established and validated clinical decision tools for cervical spine clearance. Almost all the rules include spinal tenderness on exam as an indication for imaging. Our goal was to apply GLASS, a previously derived clinical decision tool for cervical spine clearance, to thoracolumbar injuries. GLass intact Assures Safe Spine (GLASS) is a simple, objective method to evaluate those patients involved in motor vehicle collisions and determine which are at low risk for thoracolumbar injuries.

**Methods:**

We performed a retrospective cohort study using the National Accident Sampling System-Crashworthiness Data System (NASS-CDS) over an 11-year period (1998–2008). Sampled occupant cases selected in this study included patients age 16–60 who were belt-restrained, front- seat occupants involved in a crash with no airbag deployment, and no glass damage prior to the crash.

**Results:**

We evaluated 14,191 occupants involved in motor vehicle collisions in this analysis. GLASS had a sensitivity of 94.4% (95% CI [86.3–98.4%]), specificity of 54.1% (95% CI [53.2–54.9%]), and negative predictive value of 99.9% (95% CI [99.8–99.9%]) for thoracic injuries, and a sensitivity of 90.3% (95% CI [82.8–95.2%]), specificity of 54.2% (95% CI [53.3–54.9%]), and negative predictive value of 99.9% (95% CI [99.7–99.9%]) for lumbar injuries.

**Conclusion:**

The GLASS rule represents the possibility of a novel, more-objective thoracolumbar spine clearance tool. Prospective evaluation would be required to further evaluate the validity of this clinical decision rule.

## INTRODUCTION

Effective diagnosis of spinal column injuries continues to present a diagnostic challenge to clinicians both in the pre-hospital and in-hospital environment. Risks of motion or force exacerbating potential spinal injuries have been historically overstated but have led to the challenge of deciding who needs radiography to exclude significant injury to the spinal column after blunt trauma. The most well-known decision tools are the NEXUS and the Canadian cervical spine rules. Both of these well-known studies deal with cervical spine injuries, while thoracolumbar (TL) spine fractures from blunt trauma have not garnered the same attention.

Despite the lack of attention in the literature, prevalence of TL fractures is actually higher than cervical spine fractures. One study demonstrated the prevalence of TL injuries in blunt trauma patients undergoing radiographic imaging to be 6.3%^1^ compared with described prevalence of approximately 2.4% for cervical spine injuries.^2^

Several studies have attempted to identify which factors accurately identify patients who should undergo radiographic imaging.^3^,^4^ O’Connor and Walsham conducted a literature review evaluating the indications for TL imaging in blunt trauma patients.^3^ They reviewed 17 studies and came up with the following indications for imaging that would yield an anticipated sensitivity of 99.1%:

- High-risk mechanism of injury, defined as motor vehicle collision (MVC) ≥ 45mph, fall of ≥ 10 feet, ejection from motor vehicle or motorcycle, or any mechanism of injury outside of these criteria that could cause TL fracture;
- Painful distracting injury, defined as painful torso or long-bone injury sufficient to distract the patient from the pain of the TL injury;
- New neurological signs, or back pain or tenderness;
- Cognitive impairment, defined by Glasgow Coma Scale (GCS) < 15, abnormal mentation or clinical intoxication;
- Known cervical spine injury.

The indications for imaging of the cervical and TL spine in blunt trauma are similar. Neurological signs and symptoms, spinal column pain or distracting injuries, altered mental status or intoxication are commonly cited as an indication for imaging. Mechanism remains a component of many rules to identify the high-risk cohort and requires imaging even in the absence of physical findings.

The GLASS decision tool was derived in an attempt to identify those individuals involved in low-energy MVCs for whom cervical spine imaging could be excluded by looking at objective criteria at the scene of the accident rather than the subjective complaints of the patient.^5^ Given the excellent characteristics of this decision tool for cervical spine injuries (sensitivity 95%, negative predictive value 99.2%, specificity 54%) we sought to evaluate if it could also be applied to determine those patients who are at low risk of TL spine fractures in low-mechanism MVCs. This could potentially eliminate unnecessary spinal immobilization of patients who have complaints of back pain but are at extremely low risk of a TL spine fracture that would require surgical intervention. Additionally, it may decrease radiography use in the emergency department for patients with findings such as pain or intoxication, which may otherwise prompt imaging.

Our study sought to determine if the previously derived GLASS decision tool could be applied to adequately exclude TL spine fractures after MVC. The GLASS criteria are as follows:

- Patient age 16–60 years
- No damage to any of the vehicle’s windows
- No airbags deployed
- Patient was a front-seat occupant.
- Patient was restrained by a lap and shoulder belt.

**Population Health Research Capsule**

What do we already know about this issue?

The current clinical decision tools to determine which patients are at low risk of thoracolumbar injury in a motor vehicle accident have not been as robustly studied as those for cervical spine injuries. The current criteria for evaluating thoracolumbar injuries focus on mechanism of injury, painful distracting injuries, new neurological signs, back pain or tenderness, cervical spine injury, and cognitive impairment. Some of these patients may have back pain and tenderness, and thus could not be clinically cleared and require radiographic imaging.

What was the research question?

Can the previously derived GLASS (GLass intact Assures Safe Spine) Criteria be applied to patients involved in a motor vehicle accident to determine if they are at low risk for a thoracolumbar injury?

What was the major finding of the study?

Those patients who met the GLASS Criteria were found to be at low risk for any significant thoracolumbar injuries.

How does this improve population health?

Spinal injury remains a significant concern in motor vehicle collisions. The GLASS rule, if validated, may decrease unnecessary immobilization and decrease the expense and risks associated with unnecessary radiographic imaging.

### Study Design

We conducted a retrospective cohort study to evaluate the association between a low-energy MVC and the likelihood of the vehicle occupant sustaining a TL spine injury that required surgical intervention or treatment with immobilization using the GLASS criteria. We used the National Highway Traffic Safety Administration National Automotive Sampling System Crashworthiness Data System (NASS-CDS) to test the GLASS clinical decision tool. This is the same database that was used to derive the GLASS cervical spine rule. The institutional review board exempted this study from its review.

### Setting

We used data from NASS-CDS to enroll cases for the cohort study.^6^ The NASS-CDS database provides nationally representative data regarding MVCs based on a weighted annual sample of approximately 5,000 police-reported collisions.^7^ To be recorded in the database, at least one of the vehicles involved in the accident must have been damaged enough to require it being towed from the scene. NASS-CDS includes researcher-determined detailed information for each individual crash, including vehicle properties, damage to the vehicle, crash conditions, occupant characteristics and the injury outcome sustained by each vehicle occupant. A NASS field investigator measures over 200 different data points on each vehicle enrolled in the database. This includes investigating and documenting the status of all the glass on a vehicle including all windows, mirrors, etc., as well as the status of the airbag deployment. The injury severity assessment for each NASS-CDS case is done based on the Abbreviated Injury Scale (AIS) scoring system; in addition, injury description, severity rating, and identification of injury source are performed based on medical records and field investigation.^8^

### Sample Selection

We selected motor vehicle occupants between the ages of 16 – 60 involved in a collision and reported in the NASS-CDS during the years 1998 to 2008 as sample cases in the study. The selection criteria further required that the occupant had to be seated in the front driver or passenger position, with lap and shoulder belt restraint, and the vehicle had to be equipped with functional, frontal driver and passenger airbags, which did not deploy during the event of the crash. The vehicle type considered in the study was limited to passenger cars, sport utility vehicles, light trucks and vans. To adequately evaluate post-crash window integrity as an exposure measure, vehicles were pre-screened to include the ones that had intact windows and all adjustable windows in completely closed position (windows up) prior to the MVC. We excluded from the study cases of vehicle fire, water submersion and other non-representative cases (sample weighting factors in excess of one million).

### Exposure Outcomes And Measures

The exposure measure used in this analysis was the post-crash integrity of the vehicle windows. For each case selected in the sample, it was determined whether the windshield, door windows or the rear window had been damaged as a result of the crash impact. The cause of window damage could have been due either to occupant contact or contact from external sources in the crash environment. The outcome measure of the analysis was the incidence of a clinically important TL spine injury with an AIS severity magnitude of two and higher (AIS 2+) as reported in NASS-CDS. Clinically important TL spine injuries, as defined in this study, include cord contusion, cord laceration and vertebral body injury, which may include fracture, herniation, or dislocation.

### Data Analysis

Using a 2 x 2 contingency table, we analyzed the measure of association between the post-crash integrity of the vehicle windows and the outcome event of an occupant in this vehicle to sustain an AIS 2 or more severe TL spine injury. In the analysis we computed chi-square statistic to compare the probability of sustaining a TL injury for the two exposure groups considered in the study, and we reported the association measure in terms of relative risk with 95% confidence intervals (CI). The performance characteristic of a rule, which states that post-crash integrity of vehicle windows is indicative of the absence of TL spine injuries, was evaluated with 95% CI for sensitivity, specificity and negative predictive value. We performed all analyses with the SAS statistical software, version 9.1 (SAS Institute, Inc., Cary, NC).

## RESULTS

We examined a total of 14,191 occupant cases that when weighted represented over 10 million front-seat occupants involved in crashes during the study period. The vehicles involved were mostly passenger cars (62%), followed by SUVs (22%) and vans (7%). The demographics of these occupants involved in a MVC included a mean age of 34 years with 54% female ratio and 81% of them seated in the driver position (Table 1).

### Thoracic spine injuries

There were a total of 7,639 crash victims with intact windows. Four of these cases had an AIS 2 or greater thoracic spine injury. Table 2 details the injury outcome of the four subjects with thoracic spine injury who would have been missed by the rule. One of the four patients, age 55, also suffered significant injuries including an aortic injury with hemorrhage, bilateral flail chest, splenic laceration, lung laceration, liver injury, and a cerebellar injury. Other injuries not listed included extremity contusions, small lacerations, and a finger fracture. There were a total of 6,552 cases with window damage, of which 68 sustained thoracic spine injury. GLASS had a sensitivity of 94.4% (95% CI [86.3–98.4%]), specificity of 54.1%(95% CI [53.2–54.9%]), and negative predictive value of 99.9% (95% CI [99.8–99.9%]).

### Lumbar spine injuries

There were 7,639 cases with intact windows. Ten subjects in these vehicles suffered lumbar injuries of AIS 2 or greater. Table 3 details the injury outcome of those 10 subjects. The 20-year-old patient suffered a cervical strain, chest wall contusion, and subgaleal hematoma, and was given an AIS code of being unconscious for less than one hour. The 39-year-old patient had unconsciousness of unknown duration, facial skin contusion, chest wall contusion, upper extremity contusion, and lower extremity contusions. The 41-year-old patient also had cervical spine sprain. One of the 45-year-old patients suffered a cervical spine disc herniation not further specified, while the other 45-year-old patient suffered a facial skin laceration and abrasion. Of the 6,552 cases with window damage, 93 had a lumbar injury. GLASS had a sensitivity of 90.3%(95% CI [82.8–95.2%]), specificity of 54.2%(95% CI [53.3–54.9%]), and negative predictive value (NPV) of 99.9% (95% CI [99.7–99.9%]).

### Thoracic and Lumbar combined

The study characteristics when combining all thoracic and lumbar injuries with intact glass were as follows: sensitivity 92.0% (95% CI [86.9–95.5%]); specificity 54.1% (95% CI [53.5–54.7%]); and NPV of 99.9% (95% CI [98.8–99.9%]). Table 4 details a 2 x 2 contingency table for both thoracic and lumbar injuries.

## DISCUSSION

If prospectively validated, the GLASS rule would be a useful adjunct to clinical exam, which is unreliable for TL,^9^ for emergency physicians making imaging decisions for injured patients. Moreover, in combination with a validated GLASS cervical spine rule, this data could reduce the number of patients immobilized in the pre-hospital setting for spinal “precautions.” The assessment of vehicle windows and airbags is simple and rapid and typically already done by EMS providers, making the GLASS rule an ideal candidate for a prehospital clinical decision rule. More complex clinical decision rules such as the Maine Protocol have been derived and well-validated for proper use by prehospital personnel.^10^ Decreasing immobilization leaves more time to focus on clinically important care, decreases risk to providers by allowing them to move patients from dangerous traffic or scene conditions more rapidly, and increases patient comfort while reducing well-documented harms of immobilization.

This is particularly useful for emergency physicians as well because there are currently no validated rules with both good sensitivity and specificity for TL spinal injuries.^11^ Practice patterns widely vary among institution and clinicians for this reason.^12^ This mechanism-based tool would provide good confidence to providers choosing to forgo radiation from computed tomography or plain radiography after a low-risk MVC, although its poor positive predictive value would not mandate high suspicion in the absence of physical findings. A prospective study is ongoing, and if GLASS is validated it would be the first such tool for these injuries.

## LIMITATIONS

A primary limitation of the study is that we performed a retrospective cohort analysis using a national database. A prospective study would further clarify some of the injuries we found in our study group that we suspect were inaccurately reported in the database. It must be realized that the analysis performed for rule performance is not population-based and includes a random selection of police-reported cases involving tow-away crashes. This may have resulted in the slightly lower sensitivity recorded for GLASS, when compared to NEXUS and the Canadian C-Spine Rule, because we are not capturing occupants involved in accidents in which neither vehicle required towing. Although the NASS-CDS includes weighting information to extrapolate the risk measures at the national level, inaccuracies associated with the weighting scheme to appropriately address specific injury outcomes and glass-damage exposure may lead to misleading results. The NASS-CDS data used was from 1998–2008, including older-model vehicles, which may have affected the data compared to modern vehicles as crashworthiness continues to improve. It is unknown whether window breakage would increase or decrease with this; however, other factors such as development of side curtain airbags may exclude more individuals from application of this rule.

## CONCLUSION

The GLASS decision tool holds the promise to be an effective tool to safely rule out serious thoracic or lumbar spinal injury after an MVC based solely on objective criteria. In this retrospective cohort analysis, patients involved in accidents in which none of the GLASS criteria were met were very unlikely to have suffered a clinically significant spinal injury. This decision tool needs to be prospectively validated to further clarify the actual characteristics of the rule with regard to sensitivity and specificity, as well as ease of implementation, and potential cost savings. If validated, it has the potential to decrease both unnecessary immobilization and exposure to radiography.

## Figures and Tables

**Table 1:** Descriptive summary of selected cases of front-seat occupants involved in motor vehicle collisions.

	N=14,191	% (or SD)

	N (or mean)	
Occupant		
Age (years)	34.1	12.61
Sex (male)	6582.0	46.38
Stature (cm)	170.6	10.78
Mass (kg)	76.4	19.09
Seating position (driver)	11476.0	80.87
Vehicle		
Passenger car (yes)	8767.0	61.78
SUV (yes)	3164.0	22.30
Van (including minivans) (yes)	1002.0	7.06
Light truck (yes)	74.0	0.05
Injury		
Fatality (yes)	180.0	1.27
Maximum known abbreviated injury scale (AIS)	46.43	
1	5664.0	40.21
2	678.0	4.81
3	372.0	2.64
4	154.0	1.09
5	107.0	0.76
6	22.0	0.16
Unknown	549.0	3.90

*SD,* standard deviation; *SUV*, sport (or suburban) utility vehicle.

**Table 2:** Injury outcomes in GLASS*-negative patients with thoracic Injuries (number of fracture type).

Age	Thoracic injury
37	Thoracic spine fractures with or without dislocation but no cord involvement (3).
32	Vertebral body fracture with minor compression and less than 20% loss of anterior height (2).
47	Transverse process fracture.
55	Thoracic vertebral body fracture not further specified (“burst fracture”).

*GLASS*, GLass intact Assures Safe Spine clinical decision tool.

**Table 3:** Lumbar injuries among GLASS*-negative patients (number of that fracture type).

Age	Lumbar injury
20	Transverse process fractures (2).
23	Lumbar vertebral body fracture with major compression greater than 20% loss of anterior height.
24	Lumbar vertebral body fracture with minor compression less than 20% loss of anterior height.
32	Lumbar vertebral body fracture with minor compression less than 20% loss of anterior height (2). Transverse process fractures (4).
39	Vertebral body fractures not further specified (2).
40	Lumbar strain. Lumbar disc herniation not further specified.
41	Lumbar vertebral body fracture with minor compression less than 20% loss of anterior height.
45	Lumbar disc herniation not further specified.
45	Lumbar vertebral body fracture with minor compression less than 20% loss of anterior height.
54	Spinous process fractures (2).

*GLASS*, GLass intact Assures Safe Spine clinical decision tool.

**Table 4:** 2 x 2 contingency table analyzing the association between the post-crash integrity of vehicle windows and thoracolumbar spine injuries in front-seat occupants restrained by seatbelts.

Raw counts	T-spine injury AIS 2+	No T-Spine Injury AIS 2+
GLASS positive	68	6,484
GLASS negative	4	7,635
	L-spine Injury AIS 2+	No L-Spine Injury AIS 2+
	
GLASS positive	93	6,459
GLASS negative	10	7,629

*GLASS*, GLass intact Assures Safe Spine clinical decision tool.
